# Podemos Pré-Tratar Enxertos de Veia com Adiponectina para Melhorar sua Permeabilidade?

**DOI:** 10.36660/abc.20210902

**Published:** 2021-11-22

**Affiliations:** 1 Universidade Federal de São Paulo Escola Paulista de Medicina Divisão de Cardiologia São Paulo SP Brasil Seção de Lípides, Aterosclerose e Biologia Vascular, Divisão de Cardiologia, Escola Paulista de Medicina, Universidade Federal de São Paulo, São Paulo, SP – Brasil; 2 Universidade Federal de São Paulo Escola Paulista de Medicina Santana de Parnaiba SP Brasil Escola Paulista de Medicina da Universidade Federal de São Paulo – Cardiologia, Santana de Parnaiba, SP – Brasil

**Keywords:** Doença da Artéria Coronariana, Veia Safena/transplante, Adiponectina/uso terapêutico, Permeabilidade Capilar, Ratos

O enxerto de veia safena continua sendo uma opção para pacientes com doença arterial coronariana multiarterial,^1,2^ principalmente em indivíduos com diabetes tipo 2.^3^ Embora a revascularização do miocárdio deva oferecer melhores taxas de permeabilidade do que os enxertos venosos, em muitas situações, a revascularização completa não pode ser alcançada usando apenas enxertos arteriais. A falha do enxerto venoso foi associada a desfechos cardiovasculares, incluindo mortalidade.^4,5^ Portanto, há uma necessidade de melhorar a permeabilidade do enxerto de veia. Os mecanismos envolvidos na falha do enxerto incluem hiperplasia intimal, proliferação de células musculares lisas e disfunção endotelial, entre outros.^6,7^ Tem havido algumas tentativas de melhorar a preservação dos enxertos de veia safena antes da implantação.^8^

No estudo experimental de Zhou et al.,^9^ os autores utilizaram veias jugulares autólogas implantadas como enxertos de interposição carotídea em ratos Sprague Dawley. Dois grupos receberam adiponectina (2,5 *μ*g e 7,5 *μ*g) aplicada externamente à ponte de safena, suspensa em gel de Pluronic-F127 30%. Os outros dois grupos (controles) receberam veículo ou nenhum tratamento (apenas bypass).^9^ No dia 3, a proliferação celular foi significativamente menor nos enxertos tratados com adiponectina versus controle e tratados com veículo-gel, tanto na íntima quanto na adventícia, enquanto a expressão de VCAM-1 e ICAM-1 foi significativamente regulada negativamente nos enxertos de veias tratadas com adiponectina na semana quatro. Além disso, o tratamento de enxertos de veia com géis carregados de adiponectina reduziu a espessura da íntima, da mídia e da adventícia quando comparado com o controle e enxertos de veias tratadas com gel de veículo no dia 28.^9^

A adiponectina, uma adipocina secretada pelos adipócitos, é um conhecido fator homeostático que regula os níveis de glicose, o metabolismo lipídico e a sensibilidade à insulina por meio de seus efeitos antiinflamatórios, antifibróticos e antioxidantes. Esses efeitos são mediados por sua interação com dois receptores: AdipoR1 e AdipoR2. Descritos inicialmente como sendo expressos no músculo esquelético e no fígado,^10^ respectivamente, eles foram identificados no miocárdio, macrófagos, cérebro, células endoteliais, linfócitos, tecido adiposo e células beta pancreáticas.^11^ A adiponectina é um dos hormônios com as mais elevadas concentrações plasmáticas e a via da adiponectina pode desempenhar um papel crucial nos mecanismos de tratamento do diabetes mellitus tipo 2 e outras doenças afetadas pela resistência à insulina, como cânceres ou doenças cardiovasculares.^12^ Um estudo de Marino et al.,^13^ mostrou que a adiponectina foi associada com fibro ateroma de capa fina em angina estável, visto por histologia virtual.^13^ Recentemente, Chu et al.,^14^ e Gatto et al.,^15^ demonstraram que a atorvastatina pode inibir a hiperplasia intimal no modelo de enxerto de veia de rato pela inibição da via p38 MAPK. Os resultados de Zhou et al.,^9^ ampliam as possibilidades de tratamento de enxertos venosos, utilizando a via da adiponectina como alvo terapêutico para melhorar sua perviedade. No entanto, os mecanismos precisos precisam ser elucidados, e mais estudos de longo prazo em humanos são necessários para confirmar se eles se traduzem em maior permeabilidade do enxerto venoso e, consequentemente, melhores resultados.
