# Supplementary material for: Podemos Pré-Tratar Enxertos de Veia com Adiponectina para Melhorar sua Permeabilidade?
Source: Arq Bras Cardiol. 2021 Nov 22;117(6):1189–90. [Article in Portuguese] doi: 10.36660/abc.20210902 (PMC8757163; doi:10.36660/abc.20210902)
Supplement: Translation

# Translation: Can We Pretreat Vein Grafts with Adiponectin to Improve Their Patency?

**Affiliations:** 1 São Paulo SP Brazil Seção de Lípides, Aterosclerose e Biologia Vascular, Divisão de Cardiologia, Escola Paulista de Medicina, Universidade Federal de São Paulo, São Paulo, SP – Brazil; 2 Santana de Parnaiba SP Brazil Escola Paulista de Medicina da Universidade Federal de São Paulo – Cardiologia, Santana de Parnaiba, SP – Brazil

**Keywords:** Coronary Artery Disease, Saphenous Vein, Adiponectin/therapeutic use, Capillary Permeability, Rats

Saphenous vein graft remains an option for patients with multivessel coronary artery disease,^1,2^ mainly in individuals with type 2 diabetes.^3^ Although artery bypass should offer better patency rates than venous grafts, in many situations, complete revascularization cannot be achieved using solely arterial grafts. Venous graft failure has been associated with cardiovascular outcomes, including mortality.^4,5^ Therefore, there is a need to improve vein graft patency. The mechanisms involved in graft failure include intimal hyperplasia, smooth muscle cell proliferation, and endothelial dysfunction, among others.^6,7^ There have been some attempts to improve the preservation of saphenous vein grafts before implantation.^8^

In the experimental study by Zhou et al.^9^ the authors used autologous jugular veins implanted as carotid interposition grafts in Sprague Dawley rats. Two groups received adiponectin (2.5 μg and 7.5 μg) applied externally to the vein bypass grafts, suspended in a 30% Pluronic-F127 gel. The other two groups (controls) received vehicle or no treatment (bypass only).^9^ At day 3, cell proliferation was significantly lower in adiponectin-treated versus control and vehicle-gel-treated grafts, both in intima and adventitia, whereas expression of VCAM-1 and ICAM-1 was significantly down-regulated in the adiponectin-treated vein grafts in week four. In addition, the treatment of vein grafts with adiponectin-loaded gels reduced intimal, media, and adventitia thickness when compared with the control and vehicle-gel-treated vein grafts at day 28.^9^

Adiponectin, an adipokine secreted by adipocytes, is a well-known homeostatic factor that regulates glucose levels, lipid metabolism, and insulin sensitivity through its anti-inflammatory, anti-fibrotic, and antioxidant effects. These effects are mediated by its interaction with two receptors: AdipoR1 and AdipoR2. Initially described as being expressed in skeletal muscle and the liver,^10^ respectively, they were identified in the myocardium, macrophages, brain, endothelial cells, lymphocytes, adipose tissue, and pancreatic beta-cells.^11^ Adiponectin is one of the hormones with the highest plasma concentrations, and the adiponectin pathway can play a crucial role in the mechanisms to treat type 2 diabetes mellitus and other diseases affected by insulin resistance like cancers or cardiovascular diseases.^12^ A study by Marino et al.^13^ showed that adiponectin was associated with thin-cap fibro atheroma in stable angina, seen by virtual histology.^13^ Recently, Chu et al.^14^ and Gatto et al.^15^ demonstrated that atorvastatin could inhibit intimal hyperplasia in the rat vein graft model by inhibiting the p38 MAPK pathway. The results by Zhou et al.^9^ expand the possibilities of treating vein grafts, using the adiponectin pathway as a therapeutic target to improve its patency. However, the precise mechanisms need elucidation, and further long-term studies in humans are necessary to confirm if they translate into longer vein graft patency, and consequently, better outcomes.
